# Supplementary material for: Association Between Multivitamin Use on Admission and Clinical Outcomes in Patients Hospitalised with Community-Acquired Pneumonia: A Case—Cohort Study
Source: Nutrients. 2024 Nov 23;16(23):4009. doi: 10.3390/nu16234009 (PMC11643628; doi:10.3390/nu16234009)
Supplement: Supplementary file 1 [file nutrients-16-04009-s001.zip › nutrients-3307153-supplementary.pdf]

Supplementary Table S1 Covariate balance summary after inverse probability of treatment weighting (IPTW).

Covariate	Standardised Differences (Raw)	Standarised Differences (Matched)	Variance Ratio (Raw)	Variance Ratio (Matched)
Age	-0.04	-0.04	0.84	0.96
Sex	-0.07	-0.01	1.03	1.00
Charlson index	0.41	0.01	1.01	0.83
HFRS	0.12	-0.01	1.36	1.24
MUST score	0.09	0.01	1.10	0.97
CURB65	0.08	-0.03	0.84	0.83
CAD	0.09	-0.05	1.28	0.89
CKD	0.45	-0.00	1.60	0.99
Cancer	0.03	0.01	1.05	1.02
Smoking	0.11	-0.04	1.61	0.87
Alcoholism	0.14	0.03	1.65	1.09
Chronic lung disease	0.32	-0.08	1.21	0.98
IRSD	0.04	-0.03	1.17	1.24
Haemoglobin	-0.10	0.02	1.06	1.22
WBC count	0.08	-0.04	1.73	1.39
CRP	-0.04	-0.08	1.00	0.63
NLR	-0.04	-0.08	1.91	3.02
Urea	0.23	0.04	1.25	1.00
Creatinine	0.51	0.04	6.92	1.26
Albumin	0.06	0.01	0.93	0.95
INR	0.05	-0.06	1.37	1.01
Antibiotics	-0.15	-0.01	0.82	1.01

HFRS, Hospital Frailty Risk Score; MUST, Malnutrition Universal Screening Tool; CURB-65, (pneumonia severity score calculated from following parameters: confusion, urea levels >7mmol/L, respiratory rate ≥30/min, blood pressure systolic <90mm Hg or diastolic ≤60mm Hg , and age ≥65 years); CAD, coronary artery disease; CKD, chronic kidney disease; IRSD, index of relative socioeconomic disadvantage; WBC, white blood cell; CRP, c-reactive protein; NLR, neutrophil:lymphocyte ratio; INR, international normalised ratio
